# Supplementary figures and images for: Expression of Aspergillus niger glucose oxidase in Pichia pastoris and its antimicrobial activity against Agrobacterium and Escherichia coli
Source: PeerJ. 2020 Aug 4;8:e9010. doi: 10.7717/peerj.9010 (PMC7413082; doi:10.7717/peerj.9010)

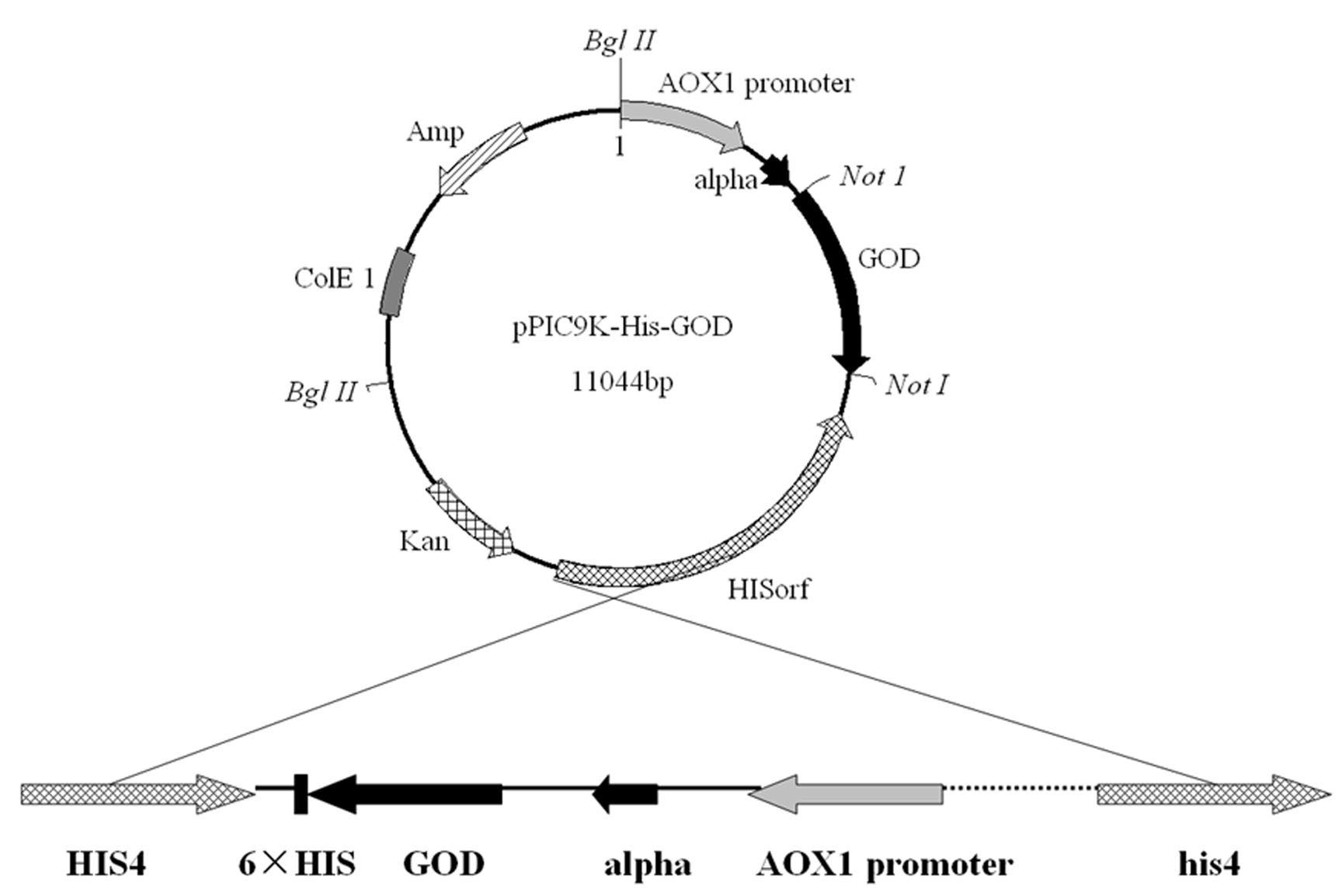

Supplement: Supplemental Information 1 — Letters in italics designate restriction endonuclease sites. GOD, the gene for Aspergillus niger glucose oxidase; alpha, ɑ-mating factor; AOX1, the promoter of alcohol oxidase 1; HISorf, the open reading frame of the histidine deaminase gene 4. The vector was linearized with Bgl II before transformation. [file peerj-08-9010-s001.jpg]
