# Supplementary material for: Expression of Aspergillus niger glucose oxidase in Pichia pastoris and its antimicrobial activity against Agrobacterium and Escherichia coli
Source: PeerJ. 2020 Aug 4;8:e9010. doi: 10.7717/peerj.9010 (PMC7413082; doi:10.7717/peerj.9010)
Supplement: Supplemental Information 2 [file peerj-08-9010-s002.docx]

sTable 1 Primers for PCR based on conserved sequences of glucose oxidase gene

| Primers | Sequences |  |
| --- | --- | --- |
| Primer 1 | 5’-GCGGCCGCCCATCATCATCATCATCATCCAGGTGGTCCAAGCAATGGCATTGAAGCCA-3’ |  |
| Primer 2 | 5’-GCGGCCGCACCACTCACTGCATGGAAGC-3’ |  |

Note: The underlined portion of P1 highlights His 6-tag with flanking Sma I site at C-terminal and N-terminal
